# Flavonoids from *Lycium barbarum* Leaves Exhibit Anti-Aging Effects through the Redox-Modulation

**DOI:** 10.3390/molecules27154952

**Published:** 2022-08-03

**Authors:** Yinhong Niu, Jiale Liao, Haitao Zhou, Chih-chen Wang, Lei Wang, Yanli Fan

**Affiliations:** 1 School of Food & Wine, Ningxia University, Yinchuan 750021, China; yhniu0909@163.com (Y.N.); ljlbbcmsn@163.com (J.L.); 2 National Laboratory of Biomacromolecules, CAS Center for Excellence in Biomacromolecules, Institute of Biophysics, Chinese Academy of Sciences, Beijing 100101, China; htzhou102@126.com (H.Z.); chihwang@sun5.ibp.ac.cn (C.-c.W.); 3 Central Laboratory, Luoyang Central Hospital Affiliated to Zhengzhou University, Luoyang 471009, China; 4 College of Life Sciences, University of Chinese Academy of Sciences, Beijing 100049, China

**Keywords:** *Lycium barbarum* leaf flavonoids extracts, oxidative stress, *Caenorhabditis elegans*, lifespan, EPO, MAPK pathway

## Abstract

*Lycium barbarum* leaves are a kind of vegetable, and modern nutrition studies have found that they have an anti-aging function. Our study aims to investigate the anti-aging effects of *Lycium barbarum* leaf flavonoid (LBLF) extracts and its underlying molecular mechanism. LBLFs were purified using D101 and polyamide resin, characterized by ultraperformance liquid chromatography coupled with mass spectrometry, and administered to hydrogen peroxide (H_2_O_2_)-treated human umbilical vein endothelial cells (HUVECs) and *Caenorhabditis elegans*. Appropriate enrichment conditions were optimized through dynamic adsorption and desorption experiments, the content of flavonoids reached 909.84 mg/g, rutin and kaempferol being the main ones. LBLFs attenuated H_2_O_2_-induced HUVEC apoptosis, decreased reactive oxygen species and malondialdehyde production levels, increased superoxide dismutase, glutathione peroxidase and catalase activities. Furthermore, pre-treatment with LBLF increased mRNA expression of erythropoietin (EPO) and heme oxygenase-1 (HO-1) via the mitogen-activated protein kinase (MAPK) signaling pathway in HUVECs. Compared with 100 µM rutin monomer, LBLF prolonged the lifespan of *Caenorhabditis elegans*, enhanced their mobility in middle life stages and upregulated expression of *sod-2*, *gcs-1* and *skn-1* genes, which indicated that the anti-aging effects of LBLF were due to its redox-modulation.

## 1. Introduction

Accompanying the improvement of food supply and the aging of the population, diseases such as cancer, cardiovascular and diabetes have been listed as the foremost dangers to human health [1]. The major risk factor for these prevalent diseases has been identified as aging [2], and oxidative stress has been associated with aging and age-related diseases. Therefore, the search for natural antioxidants to alleviate oxidative stress and slow down aging is crucial. Numerous reports have highlighted flavonoids as the most representative plant antioxidants [3]. A number of these, including quercetin and blueberry polyphenols, exhibit longevity extension properties [4].

*Lycium barbarum* (*L. barbarum*) leaves have also been commonly used as tea, vegetables, Chinese herbal medicine and are nowadays highly emphasized as a functional tea or in dietary supplements [5]. Currently, the beneficial effects of flavonoids in antioxidation, anti-inflammatory, antibacterial activity, anticancer and hepatoprotective activities are being recognized in numerous studies [6]. Flavonoids have become increasingly popular in health care because of their remarkable biochemical and pharmacological activities [7]. The active ingredients in *L. barbarum* leaves and *L. barbarum* fruit are similar, but the content of the flavonoids in the leaves is higher.

Flavonoid extracts usually contain impurities (e.g., terpenoids, polysaccharides, proteins, lignans) which severely limit their medicinal potential [8]. Therefore, it is necessary to establish an appropriate and effective method to improve the purity of flavonoids from *L. barbarum* leaves. So far, more than 19 flavonoids have been isolated from *L. barbarum* leaves [9]. Macroporous resin has been successfully applied to the isolation and purification of flavonoids from bamboo leaves [10]. Polyamide resins (RP) can be used to remove most of the impurities and enrich some chemicals [11]. This study describes an efficient method for enriching LBLF with D101 resin combined with a polyamide resin.

*L. barbarum* leaves are a new resource for future food and should be used to the greatest extent possible. Previous studies in our laboratory demonstrated that the crude extract from *L. barbarum* leaves is rich in flavonoid ingredients, and it delayed the oxidation of protein and fat in a minced-mutton compound [12]. A previous study showed that some of the extracts used in worms increased lifespans than single compounds, due to synergisms between different flavonoids [13]. However, whether *L. barbarum* leaf flavonoids (LBLFs) have anti-aging effects and what the underlying mechanism is needs further exploration. Herein, we determined the optimal extraction, separation and purification process and structure of LBLFs. By using human umbilical vein endothelial cells (HUVECs) and *Caenorhabditis elegans* (*C. elegans*) as research models, we systematically explored the redox-modulation and anti-aging effects of LBLFs.

## 2. Materials and Methods

### 2.1. Materials

*L. barbarum* leaves were purchased from Yinchuan Yuxin Wolfberry Seed Industry Co., Ltd. (Yinchuan, China) D101 resin was obtained from Anhui Samsung Resin Technology Co., Ltd. (Hefei, China) and PR column (Φ 40 mm × 140 mm) was purchased from Shanghai Yuanye Biotechnology Co., Ltd. (Shanghai, China). All chemicals were analytical grade and chromatographic chemicals were of HPLC grade. Inner salt (MTS) was purchased from Promega (Madison, USA). Reactive oxygen species (ROS, S0033S), superoxide dismutase (SOD, S0109), malondialdehyde (MDA, S0131S), glutathione peroxidase (GSH-Px, S0058), and catalase (CAT, S0051) assay kits were bought from Beyotime Biotechnology (Beijing, China). *C. elegans* and *E. coli* OP50 were provided by Professor Chang Chen, Institute of Biophysics (Beijing, China).

### 2.2. Preparation of Crude Extracts from Lycium barbarum Leaves

According to our previous crude LBLF extraction method, the details are as follows [12]. *L. barbarum* leaves were crushed in a high-speed universal SUER pulverizer (Shanghai, China) for 1–2 min and sifted through 60 meshes. The dried *L. barbarum* leaf powder was placed in a flask with 200 mL ethanol solution (70%). *L. barbarum* leaf powder was extracted by reflux of 70% aqueous ethanol at 70 °C for 2 h. The extracts were combined and evaporated to a small volume by Re-52AA rotary vaporization at 55 °C (Shanghai, China), which yielded of residue (JDG-0.2 vacuum freeze-drying testing machine, Lanzhou, China), and it was stored at 4 °C. The content of flavonoids was measured using a modified Al(NO_3_)_3_–NaNO_2_ colorimetric method [14].

### 2.3. Preliminary Purification of LBLF by D101 Rein

Two grams of D101 resin and 3.728 mg/mL of crude LBLF mixed together in a 250 mL air-tight Erlenmeyer flask, then an immersion oscillator was continuously shaken at 180 rpm for 24 h at 25 °C. After reaching adsorption equilibrium, the resins were washed by 100 mL 70% ethanol solution (*v*/*v*) in a flask. The flasks were continuously shaken (Shanghai Boxun Medical Bio-Instrument Co., Ltd.) at 180 rpm for 24 h. The adsorption rate (A), adsorption capacity (Qe) and desorption rate (D) were calculated according to the equations:

(1) Qe=(C0−Ce)×V0/W

(2) $$A=\frac{C0-\mathrm{Ce}}{C0}\times 100\%$$

(3) $$D=\mathrm{Cd}\times \frac{\mathrm{Vd}}{C0-\mathrm{Ce}}\times V0\times 100\%$$

where W, Ce, C0 and Cd are the quantity of dry resin (g), the initial, absorption equilibrium and desorption concentrations (mg/mL) of samples, respectively. V0 and Vd represent the volume of sample and desorption solution (mL).

### 2.4. Repurification and Enrichment of LBLF by PR

Into a glass column, 30 g of PR was wet-packed. Then, the extracts dissolved in ethanol were loaded onto the glass column. The concentrations of the flavonoids were 1, 2, 3, 4 and 5 mg/mL; the flow rates were set as 1, 1.5, 2, 2.5 and 3 bed volume/h (BV/h), respectively. Extracts (3 mg/mL) dissolved in ethanol were loaded onto the glass column. After complete adsorption, the 3 BV ethanol solution (40, 50, 60, 70, 80 and 90% aqueous ethanol, respectively) were used to elute (1.0, 1.5, 2.0, 2.5 and 3.0 BV/h). Each fraction eluted from the PR column was collected singly; the effluent at each flow rate was collected; and the LBLF content was determined so that the corresponding adsorption or desorption rates could be calculated. Finally, rotary evaporation was performed at 55 °C to a small volume.

### 2.5. Identification of Compounds by Ultra Performance Liquid Chromatography-Mass Spectrometry (UPLC-MS) and High Performance Liquid Chromatography (HPLC)

Electrospray ion mass spectrometer (ESI-MS) was used to scan from 100 to 1000 m/z in positive ion mode. The conditions are as follows: needle voltage 3.5 kV; electrospray ionization; mobile phase entering the mass spectrometer is split to 0.5 mL/min; capillary voltage 3.5 kV; capillary and dryer temperature 350 °C; atomizing gas (N2) pressure 40 psi; dry gas (N2) at flow rate 12.0 L/min^−1^; C18 chromatographic column (150 nm × 4.6 mm, 5 μm), eluted at 25 °C; injection volume, 10 μL; flow rate 1.0 mL/min; column temperature 30 °C; mobile phase A 0.1% FA (formic acid) + H_2_O; B, MeOH (methanol).

According to our previous research conditions [12], an HPLC system was used to measure the composition of LBLFs. Chromatographic conditions were as follows: C18 column (4.6 mm × 250 mm, 5 µm); phase A and phase B were methanol and acetic acid-water (1:100, *v/v*) solutions, respectively; injection volume 20 µL; column temperature 30 °C; UV detection wavelength 290 nm; and flow rate 1 mL/min.

### 2.6. Cell Culture and Treatment

HUVECs were cultured in Dulbecco’s Modified Eagle’s Medium (DMEM) supplemented with 10% (*v/v*) inactivated fetal bovine serum, and retained in a 5% CO_2_ incubator at 37 °C. HUVECs were grown to 70% confluence, treated with 50–800 µM H_2_O_2_ for 24 h, and then incubated at 37 °C in 5% CO_2_. LBLF in dimethyl sulfoxide (DMSO) were diluted with DMEM for the treatment of HUVECs.

### 2.7. Cell Viability Assay

The cells were incubated in 96-well plate (10^4^ cells/mL) at 5% CO_2_ incubator at 37 °C for 24 h. Control groups were cultured with 100 μL DMEM, and LBLF groups were cultured with 100 μL LBLF (10, 25, 50, 100, 200, and 400 μg/mL). After 24 h of culture, 100 μL MTS was added to each well and incubated in incubator for 2–3 h, then subjected to a microplate reader (Thermo Fisher, Waltham, MA, USA), and the average cell survival was calculated according to the following formula:

Cell viability/% = (OD of LBLF cells/OD of control cells) × 100(4)

HUVECs were inoculated in 96-well plates and cultured for 24 h, then exposed to (50, 100, 150, 200, 400, 800 μM) H_2_O_2_ for 24 h. Alternatively, HUVECs were pretreatment with LBLF (100 µg/mL) followed by exposure to H_2_O_2_ (150 μM). The cell survival was measured by MTS assay.

### 2.8. Measurement of ROS, SOD, GSH-P_x_, CAT and MDA

After treatment with LBLF or LBLF + H_2_O_2_ in 6-well plates, HUVECs were washed with PBS and then incubated with 20 μM DCFH-DA PBS at 37 °C for 15 min. The DCFH fluorescence of the cells in each well was analyzed by Becton flow cytometer (Dickinson and Company, Shanghai, China), the excitation and emission wavelengths were 488 and 525 nm, respectively. These results were expressed as a percentage of the fluorescence intensity in control group (100%).

HUVECs in the logarithmic growth phase were inoculated in 6-well plates (2 × 10^5^ cells/mL). After incubation for 24 h, LBLF (100 µg/mL) pretreatment was performed for 24 h, then H_2_O_2_ was added for 24 h. The HUVECs of each group were digested with trypsin and collected, cells homogenate was obtained by ultrasonic crushing. The activities of SOD, GSH-Px, MDA and CAT were detected in cells homogenates according to the manufacturer’s kit methods.

### 2.9. RNA-Seq Analysis

The small RNA library and sequencing were carried out by Annoroad Gene Technology Co., Ltd. (Beijing, China). In summary, total RNA was purified from mRNA using poly-T oligo-attached magnetic beads. Synthesis of first-strand cDNA, second-strand cDNA by random hexamer primers, RNase H and buffer, dNTP, DNA polymerase I, RNase H, respectively. The QiaQuick PCR kit purified the library fragments and eluted them with EB buffer. After elution, terminal repair, A-tailing and adapter addition were performed. Index-encoded samples were clustered on Illumina generation system and libraries were sequenced to produce 150 bp paired-end reads.

### 2.10. C. elegans Culture and Lifespan Assay

All *C. elegans* were cultured at 20 °C, when the plates had a great quantity of gravid adult hermaphrodites, eggs were collected using NaClO and M9 buffer. Then L1 larvae were placed onto regular plates until for L4, recorded as day 0. LBLF (200 μg/mL), rutin (100 µM), and metformin (50 mM) were dissolved in DMSO/H_2_O and added to 35 mm NGM plates to provide enough *E. coli* OP50 as food during its preparation. The control group received 0.2% (*v/v*) DMSO and 5% (*v*/*v*) H_2_O, respectively. *C. elegans* dragged out of the plate or with extruded internal organs were considered as checked [15].

### 2.11. Movement Assay

The 50 *C. elegans* individuals were placed on NGM plates with LBLF/without LBLF, and their mobility was detected on days 3, 7 and 11. The movement level of the worm was divided into three levels [16]. Three separate experiments were performed.

### 2.12. Real-Time PCR (RT-PCR) Analysis of mRNA Expression

After the treatment, the worms at Day 4 of adulthood were harvested to extract the total RNA from cells, which was performed using TRIZOL reagent kit instructions (Invitrogen, Waltham, MA, USA). RNA samples (2 µg each) were then reverse-transcribed into cDNA using HiScript II Q RT SuperMix (Vazyme, Nanjing, China). The mRNA levels were measured by reverse transcription-qPCR using RealStar Green Fast Mixture (GenStar, Beijing, China) and real-time PCR system (Applied Biosystems, Waltham, MA, USA). The samples were heated to 50 °C for 2 min, and then subjected to 40 cycles (denaturation at 95 °C for 10 min; annealing at 95 °C for 15 s; extension at 60 °C for 1 min), extension at 60 °C for 2 min (Table 1). The relative levels of each gene expression were normalized to glyceraldehyde-3-phosphate dehydrogenase (*GAPDH*) and calculated as 2^−ΔΔCT^. The primer sequences were presented in Table 2.

### 2.13. Statistical Analysis

Statistical analysis was done using one-way analysis of variance (ANOVA) using SPSS 18.0 Statistics (Chicago, IL, USA), and Origin 2018 was used for plotting of the experimental data. All results were expressed as the mean ± standard error (SE) of at least three independent experiments.

## 3. Results

### 3.1. Static Adsorption and Desorption of D101 Resin

In our laboratory, six different macroporous resins (NKA-9, AB-8, HP-20, HPD-100, DM301 and D101) have been evaluated for adsorption/desorption characteristics of flavonoids extracted from *L. barbarum* leaves, and D101 rein was selected as the most suitable one. Thus, D101 rein was used to separate and purify flavonoids, and the content of flavonoids increased from 322.0 mg/g to 868.397 mg/g.

### 3.2. Dynamic Adsorption and Desorption of Polyamide Resin

**Figure 1:**
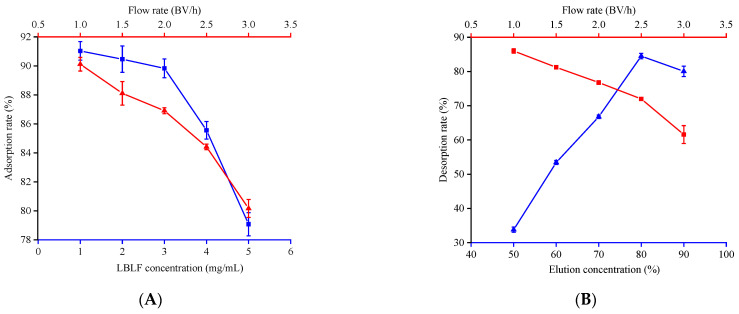

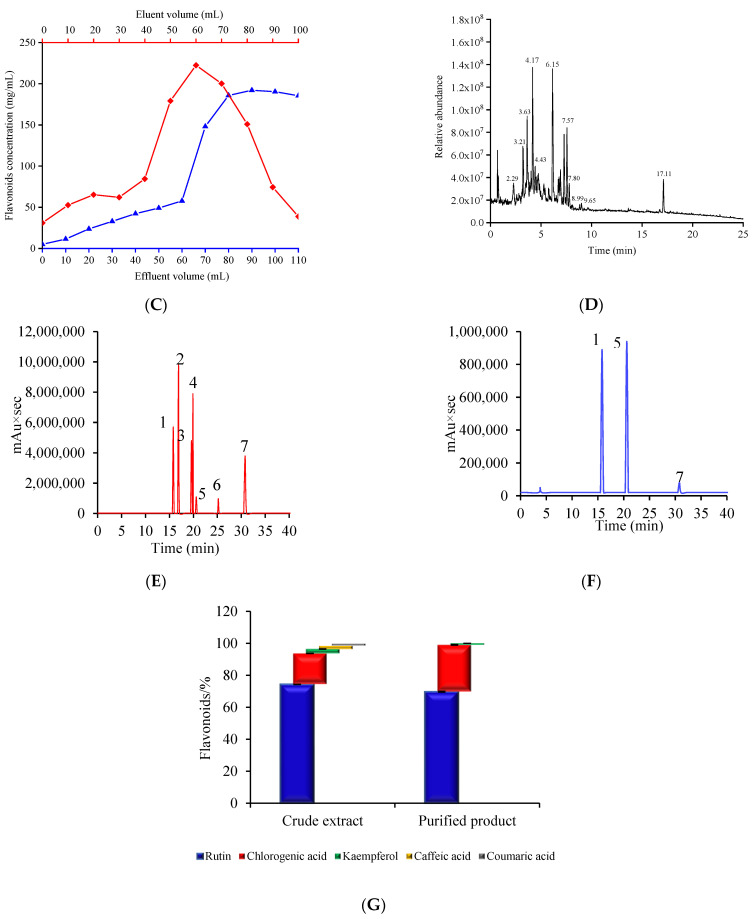
Isolation, purification and identification of LBLF. Adsorption rate of LBLF at different concentrations and flow rate (**A**). Desorption ratio of LBLF at different elution concentrations and flow rate (**B**). Dynamic adsorption and desorption curves of LBLF (**C**). The total ion chromatography of 11 flavonoids (**D**). The mixed standards (**E**) and (**F**,**G**) purified components of LBLF. 1-chlorogenic acid, 2-caffeic acid, 3-p-coumaric acid, 4-ferulic acid, 5-rutin, 6-quercetin, 7-kaempferol.

When the LBLF concentration was 3 mg/mL, the adsorption capacity reached 90%, but the capacity decreased significantly with increase concentration, probably due to the increased competition between high-concentration LBLFs on the active binding sites of the PR (Figure 1A). Flow rate was under 2 BV/h, the adsorption capacity remained at a high level. As shown in Figure 1B, the desorption rate of flavonoids reached the highest yield at 80% ethanol. The increase of LBLF flow rate had a negative impact on the impurity removal effect, and 2 BV/h was the turning point. Thus, taking efficiency into account, 3 mg/mL, 2.0 BV/h and 80% ethanol, 2 BV/h elution rate were the appropriate adsorption and desorption conditions, respectively. When the concentration of flavonoids in the effluent reaches 1/10 of the sample volume it is called the leakage point of flavonoids [17]. The dynamic LBLF adsorption curve on the PR column was “S” (Figure 1C). When the effluent volume was 60 mL (about one column volume), reaching the leak point. To determine the volume of the eluent, we decided to use 80% ethanol eluent to continuously elute the fully adsorbed PR. When the volume of the eluent was 100 mL (approximately 2.0 BV), and the flavonoids in the resin were eluted, so the sample volume and eluent volume were 1 BV and 2.0 BV, respectively.

### 3.3. Characterization of LBLF by UPLC-MS and HPLC

By using more advanced UPLC-MS chromatographic techniques, the secondary metabolites from antioxidant extracts were identified with better speed and precision [18]. They were then identified further by UPLC-MS, and 11 main monomers were obtained, including rutin, chlorogenic acid and kaempferol (Figure 1D and Figure S1, Table 3). Extracts deemed to phenolic content were analyzed by HPLC of 11 main monomer flavonoids to deliver rapid and precise quantification. According to the results of UPLC-MS analysis of LBLF, rutin, chlorogenic acid, and kaempferol with higher relative abundance were selected for HPLC quantitative analysis (Figure 1E,F). The content of flavonoids in the crude after passing through D101 reached 83.89%, which was 2.19 times that before the column. The PR column was repeatedly eluted to obtain a product with a purity greater than 90%.

As shown in Figure 1G, the highest content of rutin in the purified LBLF was 521.03 mg/g (69.71%), followed by chlorogenic acid at 219.29 mg/g (29.34%), and the lowest was kaempferol 7.06 mg/g (0.95%).

### 3.4. LBLF Alleviates H_2_O_2_-Induced Cell Death in HUVECs

**Figure 2:**
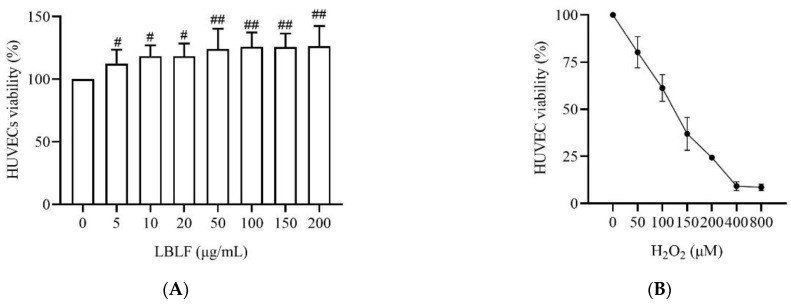

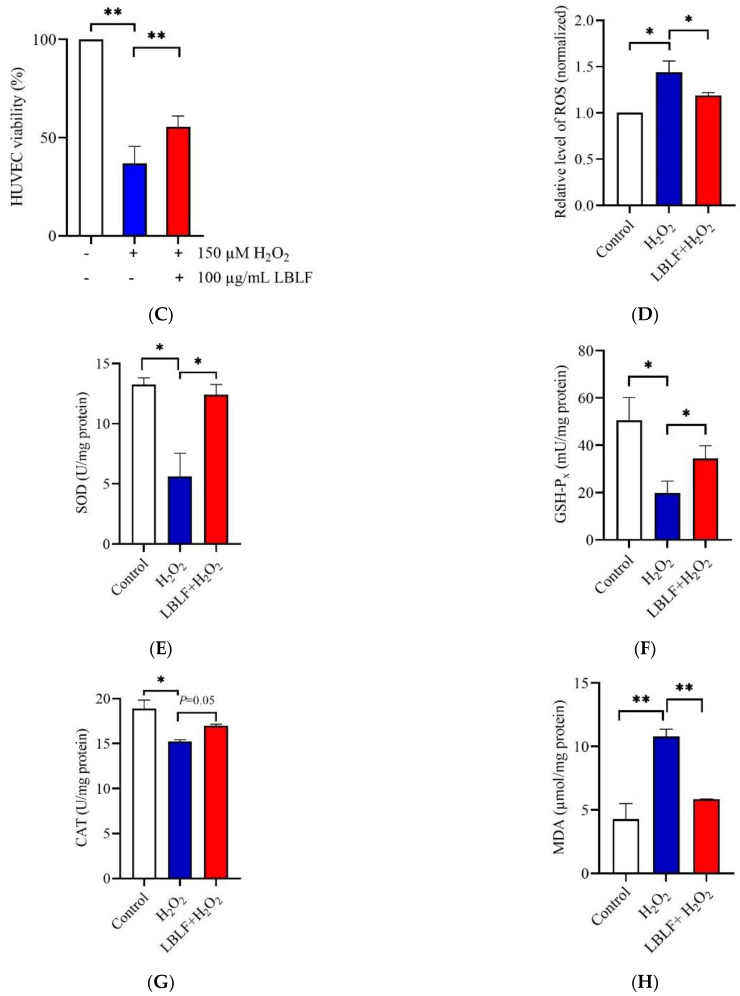
LBLF inhibits H_2_O_2_-indued injury in HUVECs by MTS assay. Effect of LBLF/H_2_O_2_ on the proliferation of HUVECs (**A**, **B**). Survival rate of H_2_O_2_-induced injury following treatment with LBLF (**C**). Effects of LBLF on ROS (**D**), SOD (**E**), GSH-Px (**F**), CAT (**G**) and MDA (**H**) levels in H_2_O_2_-injured HUVECs. Values are presented as means ± SE, n = 3, # *p* < 0.05, ## *p* < 0.01 compared to the control group, * *p* < 0.05, ** *p* < 0.01 compared to the 150 μM H_2_O_2_ group. White, blue, and red represent the control group (CK), 150 μM H_2_O_2_ treatment group (HP), and 150 μM H_2_O_2_ and 100 µg/mL LBLF cotreated group (LBLF), respectively.

We first assessed the effects of LBLF on H_2_O_2_-induced cell death in HUVECs. At the concentration range of 5–20 μg/mL LBLF, the survival of HUVECs was significantly increased compared with control group (*p* < 0.05). At a concentration range of 50–200 μg/mL LBLF, the survival of HUVECs was more significantly increased compared with control group (*p* < 0.01, Figure 2A). Therefore, 100 μg/mL were selected as the optimal concentration of LBLF treatment. The results showed that treatment with 50–800 μM H_2_O_2_ for 24 h decreased the survival of HUVECs in a concentration-dependent manner. 150 μM H_2_O_2_ decreased the survival of HUVECs to 50–60% (*p* < 0.01, Figure 2B). Therefore, 150 μM H_2_O_2_ was used to produce sufficient injury to HUVECs in the following study. To assess the effects of LBLF on H_2_O_2_-induced cell death, HUVECs were pretreated with 100 μg/mL LBLF for 24 h before exposure to 150 μM H_2_O_2_. The cell survival was significantly decreased in H_2_O_2_-treated group, whereas the survival of HUVECs pretreated with LBLF was significantly higher (*p* < 0.01, Figure 2C).

### 3.5. LBLF Suppressed H_2_O_2_-Induced Oxidative Stress Generation

The increase in fluorescence intensity resulting from the oxidation of DCFH-DA was used to indicate intracellular free radicals. Intracellular ROS levels in H_2_O_2_-treated HUVECs were significantly increased compared to control group (*p* < 0.05, Figure 2D), whereas fewer ROS were detected in the LBLF intervention group (*p* < 0.05). This indicated that LBLF can reduce the amount of H_2_O_2_-induced ROS increases in HUVECs, thereby alleviating oxidative stress and reducing the oxidative damage produced by ROS.

We then measured the effects of LBLF on SOD, GSH-Px, CAT, and MDA levels in HUVECs. Compared with control group, the activities of SOD, GSH-Px, and CAT were significantly decreased in the H_2_O_2_ group (*p* < 0.05, Figure 2E,G), indicating that the redox-modulation of HUVEC cells was damaged. However, after pretreatment with 100 µg/mL LBLF, the activities of SOD, GSH-Px, and CAT were markedly increased (*p* < 0.05) compared with the H_2_O_2_ group. Compared with the control group, the contents of MDA supernatants were increased in the H_2_O_2_ group (*p* < 0.01, Figure 2H). Compared with the H_2_O_2_ group, MDA content decreased after pretreatment with 100 µg/mL LBLF (*p* < 0.01). Altogether, these results suggested that LBLF suppressed H_2_O_2_-induced oxidative stress in HUVECs.

### 3.6. Differential Gene Expression in H_2_O_2_ and LBLF Treated Cells

**Figure 3:**
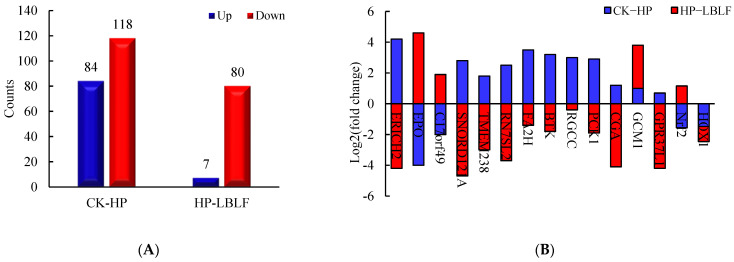

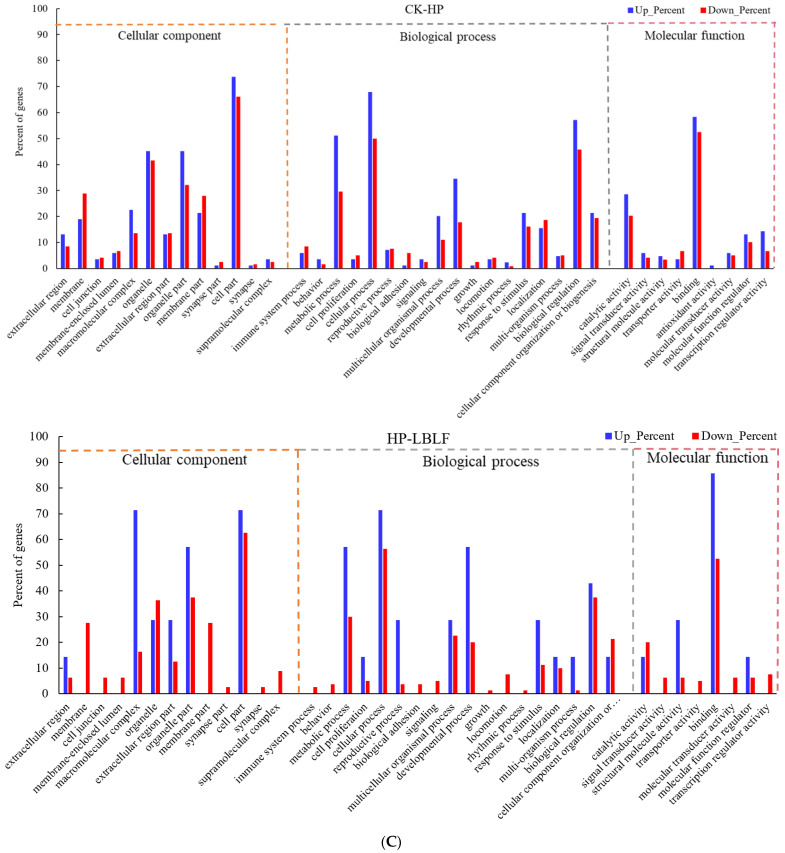
Enrichment of genes differentially expressed between different groups using RNA sequencing (**A**). The upregulated and downregulated genes that are significantly different between H_2_O_2_ and H_2_O_2_/LBLF treated HUVECs (**B**). Gene ontology (GO) function classification of the corresponding genes with differentially expressed genes in HUVECs exposed to H_2_O_2_/LBLF (**C**). The top GO enrichment of the differentially expressed genes among control group (CK), 150 μM H_2_O_2_ (HP) and 150 μM H_2_O_2_ and 100 µg/mL LBLF cotreated group (LBLF).

Next, we performed RNA-seq to compare the gene expression in H_2_O_2_ and LBLF treated cells. A total of 289 differentially expressed genes were predicted in CK-HP (control vs. 150 µM H_2_O_2_ treatment) and HP-LBLF (150 µM H_2_O_2_ vs. 100 µg/mL LBLF treatment) groups, respectively. Among these, 202 were significantly differentially expressed between the CK and HP groups (of which 84 were upregulated and 118 were downregulated). There were 87 genes that were differentially expressed (of which 7 were upregulated and 80 were downregulated) between the HP and LBLF groups (Figure 3A). From the results of the enrichment of the Gene Ontology (GO) pathway, erythropoietin (EPO), nuclear factor erythroid 2-related factor 2 (NFE2L2 or Nrf2) and heme oxygenase-1 (HO-1) were found to be differentially expressed genes involved in oxidative stress related pathways, between CK-HP and HP-LBLF groups (Figure 3B). The analysis of differentially expressed genes in biological processes revealed important GO pathways related to oxidative stress, such as metabolic process, cellular process, multicellular organismal process, developmental process, biological regulation, and response to stimuli (Figure 3C, Table S1). In addition, 4 candidate differentially expressed genes were selected and analyzed using RT-PCR. These results revealed that the expression profile of differentially expressed genes in different groups was consistent with the results from RNA-seq in response to oxidative stress.

### 3.7. Increase of EPO and HO-1 Expression Is MAPK Dependent

**Figure 4:**
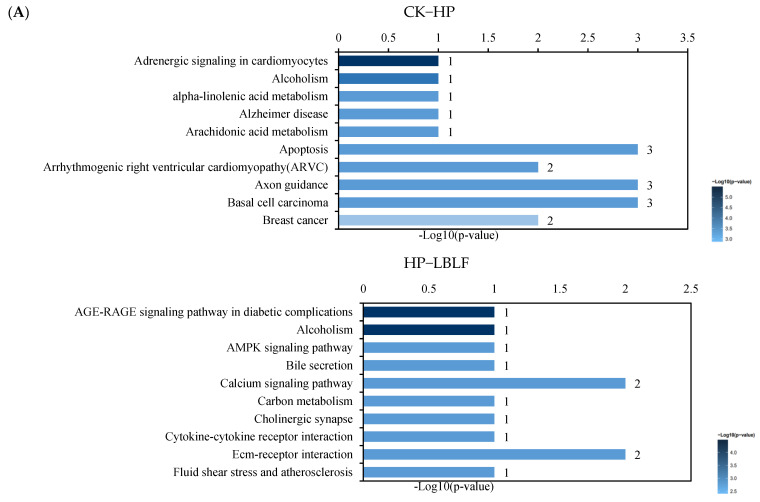

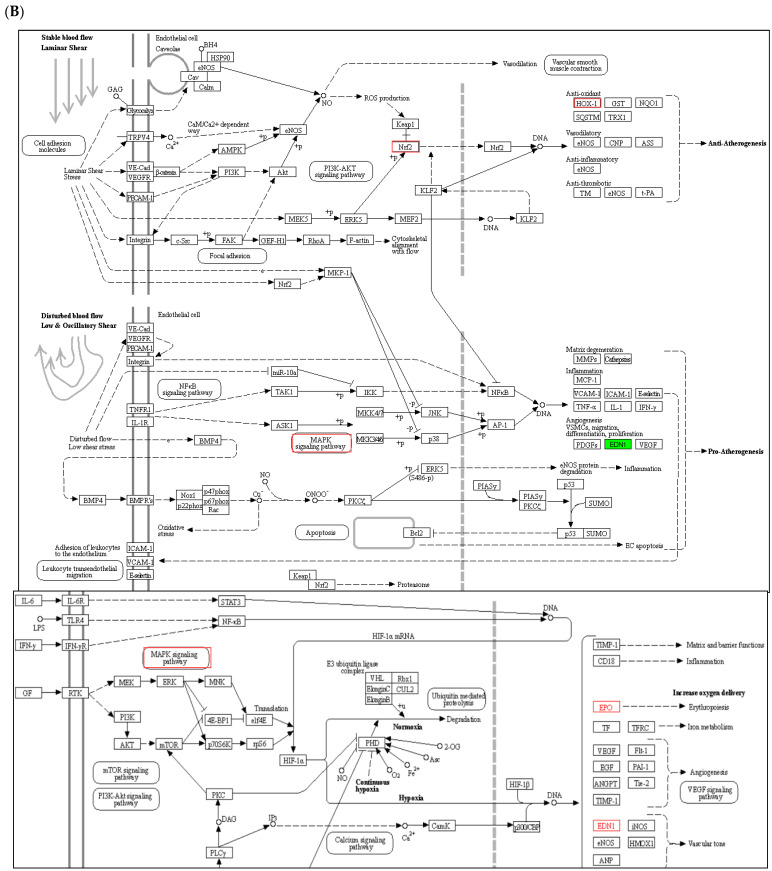
*Kyoto Encyclopedia of Genes and Genomes* (KEGG) of differentially expressed genes between different groups using RNA sequencing. The top 10 ranging KEGG pathways of the differentially expressed genes (**A**). The representative maps in KEGG analysis of the corresponding genes with differentially expressed genes exposed to H_2_O_2_/LBLF (**B**). Bar graphs CK-HP and HP-LBLF showing the KEGG terms for the differentially expressed genes among control group (CK), 150 μM H_2_O_2_ (HP), and 150 μM H_2_O_2_ and 100 µg/mL LBLF-cotreated group (LBLF).

**Figure 5:**
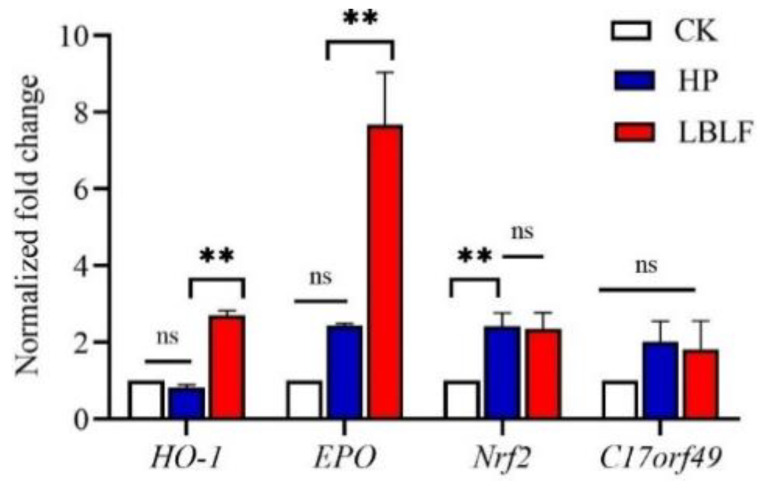
Validation of RNA-Seq data by RT-PCR. ** in the Figure represents extremely significant differences in expression levels at different stages (*p* < 0.01). Lowercase letters ns in the Figure represent unsignificant differences in expression levels at different stages (*p* > 0.05). Three RT-PCR analyses were conducted with each of three independent biological replicates and the data were analyzed using one-way analysis of variance (ANOVA). White, blue, and red represent the control group (CK), 150 μM H_2_O_2_ treatment group (HP), and 150 μM H_2_O_2_ and 100 µg/mL LBLF cotreated group (LBLF), respectively.

KEGG analysis showed that the HP-LBLF treatment groups may exhibit oxidative stress such as MAPK signaling pathways, diabetic complication pathway, and atherosclerosis (Figure 4, Table S1). Activation of MAPK-activated protein kinases mediates essential cellular processes such as survival, motility, proliferation and stress response [19]. According to the KEGG signaling pathway analysis, LBLF may protect HUVECs from oxidative stress induced by H_2_O_2_ through the MAPK signaling pathway (Figure 4B). MAPK cascades have been revealed to be associated with HO-1 activation [20], and HO-1 activity is a component of cellular defense against oxidative stress [21]. HO-1 has been revealed to exert antioxidant and anti-inflammatory effects in cardiovascular diseases induced by oxidative stress [22]. Meanwhile, the expression level of *HO*-*1* gene in the downstream of the MAPK pathway was significantly increased in H_2_O_2_ and LBLF cotreated groups (*p* < 0.01), but there was no significant difference in H_2_O_2_ treatment, compared with the control group (Figure 5). Furthermore, LBLF, like most flavonoids, activates target genes through the MAPK pathway to alleviate oxidative stress.

EPO inhibits apoptosis and promotes endothelial cell proliferation and differentiation [23]. Compared with the control group, the expression level of the *EPO* gene increased in the H_2_O_2_ and LBLF cotreated group (*p* < 0.01) (Figure 5), which was consistent with RNA-seq analysis, which found that the expression level of *EPO* gene increased 25 times (Figure 3B). We also considered whether *EPO* affected the transcription and activation of *Nrf2*, a crucial transcription factor of cellular antioxidant defense system, or the mRNA expression of *HO*-*1*. *EPO* induces *HO*-*1* expression through the activation of the MAPK (Figure 4B, Tables S1 and S2) and *Nrf2* signaling pathways, and those may be a novel mechanism for cytoprotective responses elicited by *EPO*.

### 3.8. Effect of LBLF on the Lifespan of C. elegans

**Figure 6:**
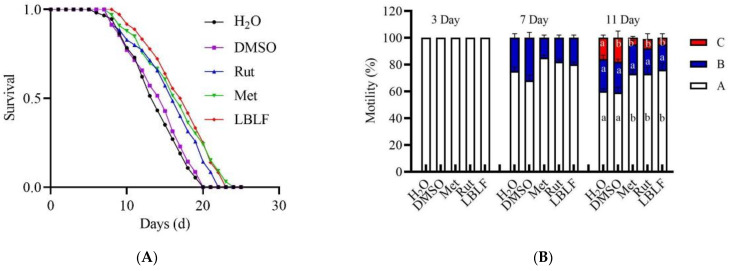

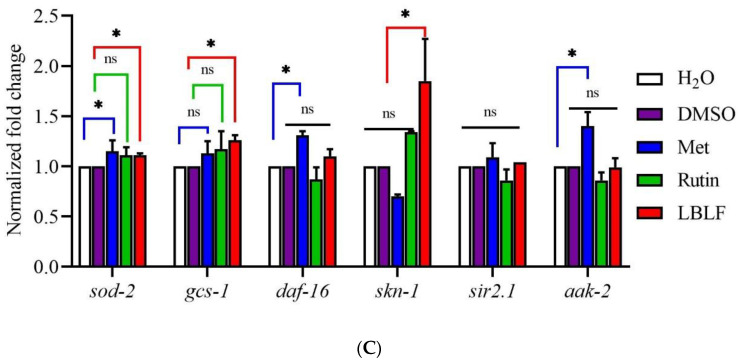
Effect of LBLF on the lifespan, motility scoring statistics and mRNA level of *C. elegans*. Effect of LBLF on the lifespan of *C. elegans* (**A**). Effect of LBLF on the motility scoring statistics of *C. elegans* (**B**). Effect of LBLF on the mRNA expression levels of related redox-modulation and anti-aging genes in *C. elegans* (**C**). Motion level was divided into three levels: if the worm moved spontaneously and smoothly, it was marked as “A”, if the worm moved in a non-sinusoidal trajectory after stimulation, it was marked as “B”, if the animal did not move forward but could respond to touch by swinging its head or tail, it was marked as “C”. Rut (100 µM rutin), met (50 mM metformin) and LBLF (200 µg/mL LBLF). At least 3000 worms were used in each group and the experiments were independently performed three times. The different letters in the column indicated significant differences in values (*p* < 0.05). * in the Figure represents extremely significant differences in expression levels at different stages (*p* < 0.05). Lowercase letters ns in the Figure represent unsignificant differences in expression levels at different stages (*p* > 0.05).

Next, we determined to study the protective effects of LBLF in an animal model. *C. elegans* has been widely used as a model for studying aging. At 20 °C, the average and maximum lifespans of *C. elegans* are approximately 15 days, 22 days, respectively [13]. *C. elegans* were cultured under different treatments, and the survival curves were monitored and the mean lifespans of *C. elegans* were calculated (Figure 6A, Table 4). The mean lifespans of *C. elegans* in the rutin and LBLF groups were 16.0 ± 0.3 days and 17.1 ± 0.4 days, compared with the DMSO group 14.9 ± 1.0 days. Thus, rutin and LBLF significantly increased the worm’s lifespan by approximately 7.6 and 15.0%, respectively (*p* < 0.001). Plant extracts increased worm lifespan at lower concentrations than single compounds [24]. Kampkötter et al. reported that quercetin (100 μM) can prolong the lifespan of C. elegans by 15%, and myricetin (100 μM) can prolong the lifespan of *C. elegans* by 18% [25,26]. Interestingly, the *C. elegans* longevity effect of 200 µg/mL from LBLF is significantly superior than the flavonoid monomer rutin.

### 3.9. Effect of LBLF on the Movement of C. elegans

The ability of *C. elegans* to perceive and react to stress is associated with aging [27]. Mobility is a measure of muscle integrity and is directly related to the physiological function of *C. elegans* [28]. Meanwhile, we tested the movement levels of *C. elegans* at three developmental stages: on day 3 (early), 7 (early-middle) and 11 (middle). On day 7, there were still 80.0% of *C. elegans* cultured with 200 μg/mL LBLF that belonged to Class A, but 82.0% and 85% of the positive control group (rutin and metformin) were still in Class A (Figure 6B). On day 11, there were still 76.0% of *C. elegans* cultured with 200 μg/mL LBLF that belonged to Class A, but 59.0% of the control group was as usual in Class A. Our study showed that LBLF significantly enhanced the age-related mobility of *C. elegans* (*p* < 0.05), especially during middle-life stages.

### 3.10. LBLF Extended Lifespan by Increasing Gene Expression

SODs act as an enzymatic antioxidant to remove superoxides [29]. With stress stimulation, SKN-1 (homolog of Nrf2) proteins are activated to regulate downstream target genes, of which *gcs-1* plays an important role in oxidative stress and aging [30]. As shown in Figure 6C, *C. elegans* were treated with LBLF at a concentration of 200 µg/mL, the levels of *sod-2*, *gcs-1* and *skn-1* mRNA were significantly increased (*p* > 0.05). The positive group (metformin) significantly upregulated (*p* > 0.05) the expression level of *sod-2* and had no significant difference between the levels of *gcs-1* and *skn-1* mRNA. However, the positive group (rutin) had no effect on the *sod-2*, *gcs-1* and *skn-1* mRNA levels. The transcription factor DAF-16 (a homolog of FOXO protein) plays a key role in stress response and longevity [31]. When *C. elegans* were treated with 200 µg/mL LBLF, the expression level of *daf-16* was not noticeably increased compared to control. The positive group (metformin) significantly upregulated the expression level of *daf-16* (*p* > 0.05), but rutin had no effect on the expression level of *daf-16*. *Sir2.1* can regulate lifespan, and the overexpression of *sir2.1* was shown to play a vital role in longevity and resistance to oxidative stress in *C. elegans* [32]. The LBLF and positive groups (metformin and rutin) did not significantly up-regulate the expression level of *sir2.1*. Similarly, *aak-2* was upregulated by 50 mM metformin treatment (*p* < 0.05), but did not significantly increase after LBLF and rutin treatment. Therefore, we proposed that LBLF alleviates oxidative stress and delays aging by upregulating the expression levels of *sod-2*, *gcs-1*, and *skn-1*.

## 4. Discussion

At present, *L. barbarum* leaves, tea, and beverages are mainly developed with *L. barbarum* leaves as raw materials. However, the medicinal value and nutritional health benefits of *L. barbarum* leaves are largely unknown. Preliminary laboratory studies have shown that the LBLF has higher in vitro antioxidant activity [33]. After *L. barbarum* leaf powders were passed through 60 meshes, the static adsorption and desorption time of D101 resin were 24 h. The influences of initial flavonoids concentration, solution flow rate, solution volume, ethanol concentration, ethanol flow rate, and ethanol volume were also studied by PR dynamic adsorption or desorption method. The optimal adsorption conditions for crude LBLF were that the sample solution volume was 2 BV, the initial concentration was 3 mg/mL and flow rate was 2 BV/h. The optimal desorption parameters were 2 BV, 80% ethanol, and a flow rate of 2.5 BV/h. Under the optimum conditions, the contents of flavonoids in the purified products were approximately 2.4 times higher than the crude extract after D101 and PR resin. Jiang et al. used polyamide resin and D101 resin adsorption resin to purify fenugreek flavonoids [34]. After separation and purification, the content of flavonoids in *L. barbarum* leaves reached 909.84 mg/g, of which rutin, chlorogenic acid, and kaempferol were the main ones. In line with our study, Mocan et al. have quantified 11 flavonoids in *L. barbarum* and *L. barbarum* leaves and the dominant compound found in both species was rutin [35].

**Figure 7:**
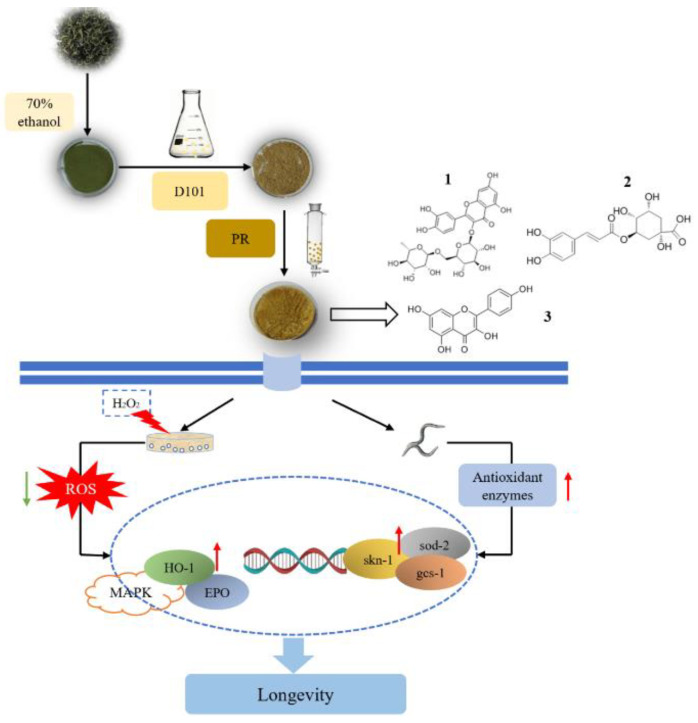
Anti-oxidation and anti-aging molecular mechanism of LBLF. 1-rutin, 2-chlorogenic acid, 3-kaempferol.

As shown in Figure 7, the effects of resin purification on the content and bioactivity of LBLF were systematically evaluated. Our modified resin increased the yield significantly and retained the antioxidant activities (in HUVECs and *C. elegans*) of LBLF in comparison to the traditional resin purification methods. LBLF increased the survival of HUVECs induced by H_2_O_2_ to protect HUVECs from oxidative stress damage, extended the lifespan of *C. elegans* by 15%, and improved the motility of *C. elegans* in the middle life stages. Interestingly, we found that LBLF was very effective at blocking ROS accumulation, increasing the activity of antioxidant enzymes (as SOD, GSH-Px and CAT), and reducing MDA levels in HUVECs. Furthermore, LBLF upregulates the expression of antioxidant enzyme genes (*sod-2*, *gcs-1* and *HO-1*) and longevity genes (*daf-16*, *skn-1*), especially *skn-1*. The redox-modulation effect of LBLF acting through the MAPK pathway might be the underlying mechanism of the anti-aging effect.

Several studies investigated the antioxidant properties of flavonoids associated with MAPK pathway. For example, quercetin protects hepatocytes from oxidative stress by activating HO-1 through the MAPK signaling pathways [36]. Epigallocatechin-3-Gallate reduces inflammation in various cells by exerting anti-MAPK activity [37]. Li et al. confirmed that rutin delayed senescence induced by oxidative stress, and the protective mechanism may be through the inhibition of oxidative stress [38,39]. Research shows the redox-modulating ability of the main chemical constituents of polyphenols (rose essential oil and *Scenedesmus obliquus*) [40,41]. Interestingly, the protective effect of LBLF was more pronounced than monomeric rutin, which might be due to synergisms among different flavonoids [13]. In summary, the present investigation revealed the marvelous potential of LBLF in antioxidant defense. Moreover, future investigations can be conducted to advance in-depth mechanistic insights and to provide support for antioxidant products.

## 5. Conclusions

After the extraction process, D101 resin and polyamide resin adsorption/desorption were applied to enrich and purify LBLF. Under optimized conditions, the LBLF compositions were rutin, chlorogenic acid and kaempferol. The oxidative stress alleviated by LBLFs was associated with the redox-modulation of ROS, SOD, GSH-Px, CAT, MDA levels and increased expression of *EPO*, *HO-1* through MAPK signaling pathway. LBLF increased the mean lifespan and improves the mobility of *C. elegans*. Interestedly, LBLF was superior to the rutin monomer in prolonging the lifespan of *C. elegans*. Moreover, LBLF prolonged lifespan by upregulating expression of *sod-2*, *gcs-1*, and *skn-1*. Thus, we found that LBLFs are advantageous natural antioxidant materials to prevent oxidative stress and slow down aging. Our study provided a scientific and theoretical basis for industrial research and development of functional *L. barbarum* leaf foods.

## Figures and Tables

**Table 1:** Reaction system of quantitative real-time PCR.

	System Name	Volume		
Ⅰ	Reverse transcription system			
	5 × HiScript II Q RT SuperMix	4 μL		
	RNA	1 μg		
	RNase-free H_2_O	Up to 20 μL		
	50 °C 15 min 85 °C 5 s 4 °C 5 min			
Ⅱ	Real-time PCR			
	2 × RealStar Green Fast Mixture (GenStar)	10 μL		
	Upstream primer	0.5 μL (10 μM)		
	Downstream primer	0.5 μL (10 μM)		
	Revers e transcript	2 μL		
	DEPC H_2_O	7 μL		
	50 °C	2 min		
	95 °C	10 min	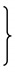	
	95 °C	15 s		40 cycles
	60 °C	1 min		
	95 °C	15 s		
	60 °C	1 min		
	95 °C	30 s		
	60 °C	15 s		

**Table 2:** Sequences of primers used for RT-PCR.

	Gene Name	Primer Sequence	
		Forward (5′–3′)	Reverse (5′–3′)
Human	*EPO*	GGAGGCCGAGAATATCACGAC	CCCTGCCAGACTTCTACGG
	*C17ORF49*	GAAACAGAAGGCTGATGTGACACT	CCTTCAATATCCACCACGTCACT
	*HO-1*	AAGACTGCGTTCCTGCTCAAC	AAAGCCCTACAGCAACTGTCG
	*NRF2*	TCAGCGACGGAAAGAGTATGA	CCACTGGTTTCTGACTGGATGT
	*GAPDH*	TCCAAAATCAAGTGGGGCGA	AAATGAGCCCCAGCCTTCTC
*C. elegans*	*sod-2*	AGCTTTCGGCATCAACTGTC	AAGTCCAGTTGTTGCCTCAAGT
	*gcs-1*	GTGCAAGTGTCGACGATCGTAC	GCGAATATGTTTTGCCAGTGGCTC
	*daf-16*	TCCTCATTCACTCCCGATTC	CCGGTGTATTCATGAACGTG
	*sir2.1*	TGGCTGACGATTCGATGGAT	ATGAGCAGAAATCGCGACAC
	*aak-2*	TGCTTCACCATATGCTCTGC	GTGGATCATCTCCCAGCAAT
	*skn-1*	ACAGGGTGGAAAAAGCAAGG	CAGGCCAAACGCCAATGAC
	*act-2*	CCCACTCAATCCAAAGGCTA	GGGACTGTGTGGGTAACACC
	*gapdh*	TCAAGGAGGAGCCAAGAAGG	CAGTGGTGCCAGACAGTTG

**Table 3:** Identification of the composition of LBLF.

No.	Retention Time (min)	[M+H]^+^	Compounds	Intensity
1	2.29	251.14	Chrysin	6,796,006
2	3.21	293.16	Angelicain	335,873.3
3	3.63	355.1	Neochlorogenic acid	32,480,266
4	4.17	472.24	Quercetin-3-*O*-glucuronide	40,736,980
5	4.43	512.24	Isochlorogenic acid A	3,446,140
6	6.15	610.19	Rutin	45,838,012
7	7.57	453.34	Taxifolin 7-rhamnoside	20,721,940
8	7.8	509.89	Isochlorogenic acid B	5,564,009
9	8.99	420.22	Daidzin	2,764,151.5
10	9.65	279.09	Kaempferol	15,970,930
11	17.11	149.02	Piperonone	11,459,642

**Table 4:** Statistical analysis of *C. elegans* lifespan.

Treatment	I	II	III	IV	V
	H_2_O	Met	DMSO	Rutin	LBLF
Mean lifespan	14.8± 0.1	15.9 ± 0.9	14.9 ± 1.0	16.0 ± 0.3	17.1 ± 0.4
Fold increase		7.5%		7.6%	15.0%
*p* value	/	I vs. II 0.13	/	III vs. IV 0.12	III vs. V <0.0001
Uncensored/n	78/100	72/100	85/100	76/100	78/100

Dimethyl sulfoxide (DMSO), rutin (Rut), metformin (Met), *Lycium barbarum* leaves flavonoids (LBLF). Compared with group I, *p* < 0.05, *p* < 0.01; compared with group II, *p* < 0.05, *p* < 0.01.

## Data Availability

Data are contained within the article or Supplementary Materials.

## Supplementary Materials

The following are available online at https://www.mdpi.com/article/10.3390/molecules27154952/s1, Figure S1 Base peak chromatograms of ingredients from LBLF by UPLC-MS in ESI+ (A), and the MS spectra of peak (B). Table S1. KEGG term and pathway enrichment analysis of differentially expressed genes between 150 μM H_2_O_2_ at 24 h treatment and control/LBLF.
