# Supplementary material for: Flavonoids from Lycium barbarum Leaves Exhibit Anti-Aging Effects through the Redox-Modulation
Source: Molecules. 2022 Aug 3;27(15):4952. doi: 10.3390/molecules27154952 (PMC9370597; doi:10.3390/molecules27154952)
Supplement: Supplementary file 1 [file molecules-27-04952-s001.zip › molecules-1811424-supplementary.pdf]

molecules

Supplementary Materials

Flavonoids from *Lycium barbarum* Leaves Exhibit Anti−Aging Effects through the Redox−Modulation

Yinhong Niu [1,2], Jiale Liao [1], Haitao Zhou [2,3], Chih−chen Wang [2,4], Lei Wang [2,4,*] and Yanli Fan [1,*]

1 School of Food & Wine, Ningxia University, Yinchuan 750021, China; yhniu0909@163.com (Y.N.); ljlbbcmsn@163.com (J.L.)
2 National Laboratory of Biomacromolecules, CAS Center for Excellence in Biomacromolecules, Institute of Biophysics, Chinese Academy of Sciences, Beijing 100101, China; htzhou102@126.com (H.Z.); chihwang@sun5.ibp.ac.cn (C.−c.W.)
3 Central Laboratory, Luoyang Central Hospital Affiliated to Zhengzhou University, Luoyang 471009, China
4 College of Life Sciences, University of Chinese Academy of Sciences, Beijing 100049, China
* Correspondence: wanglei@ibp.ac.cn (L.W.); fanyanli@nxu.edu.cn (Y.F.)

(A)

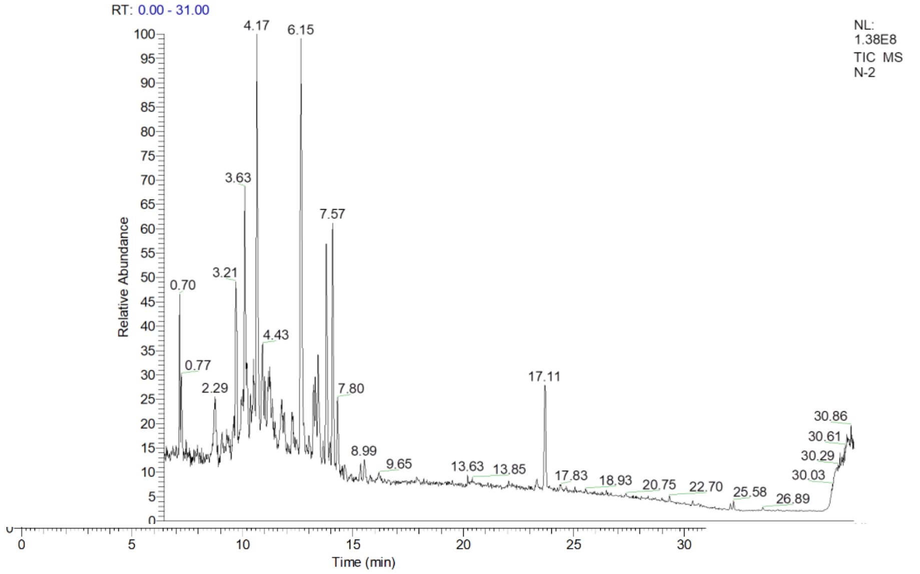
RT: 0.00 - 31.00
NL: 1.38E8
TIC MS
N-2
Relative Abundance
Time (min)
0.70
0.77
2.29
3.21
3.63
4.17
4.43
6.15
7.57
7.80
8.99
9.65
13.63
13.85
17.11
17.83
18.93
20.75
22.70
25.58
26.89
30.03
30.29
30.61
30.86

(B)

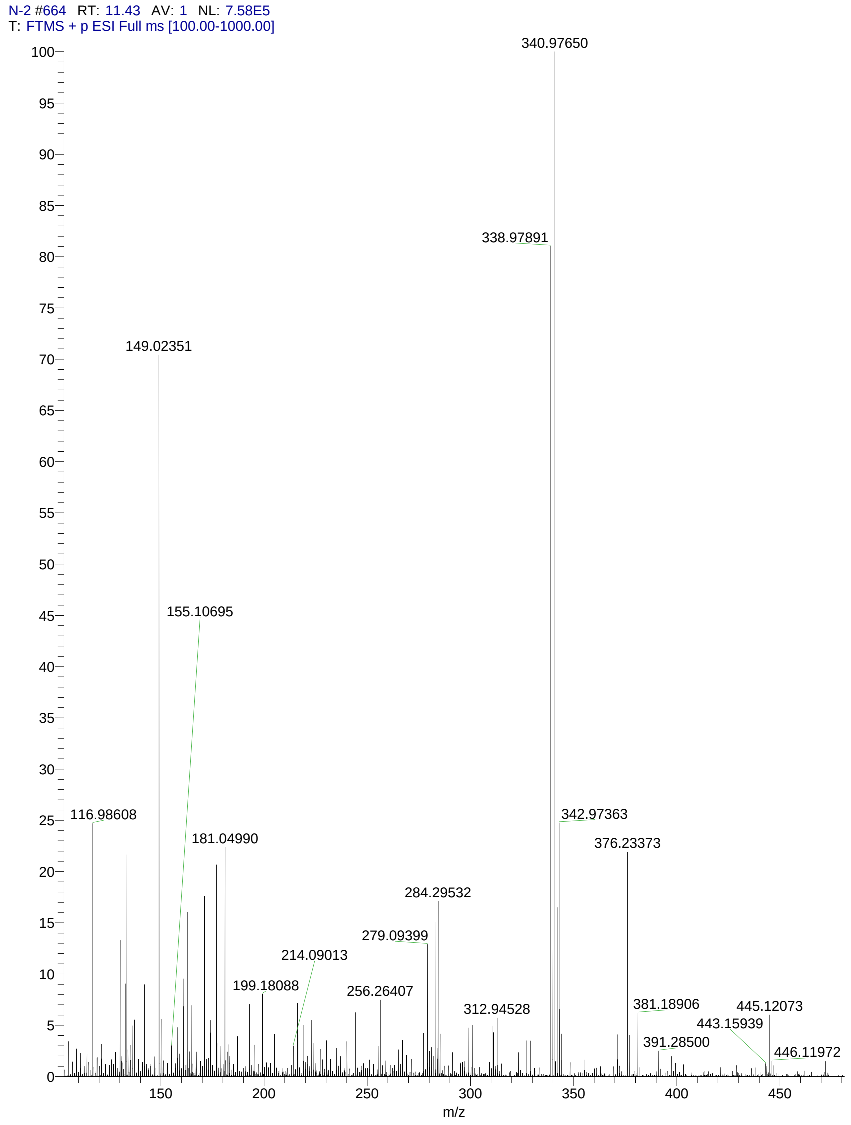
N-2 #664 RT: 11.43 AV: 1 NL: 7.58E5
T: FTMS + p ESI Full ms [100.00-1000.00]
m/z
340.97650
338.97891
149.02351
155.10695
116.98608
181.04990
342.97363
376.23373
284.29532
279.09399
214.09013
199.18088
256.26407
312.94528
381.18906
445.12073
443.15939
391.28500
446.11972

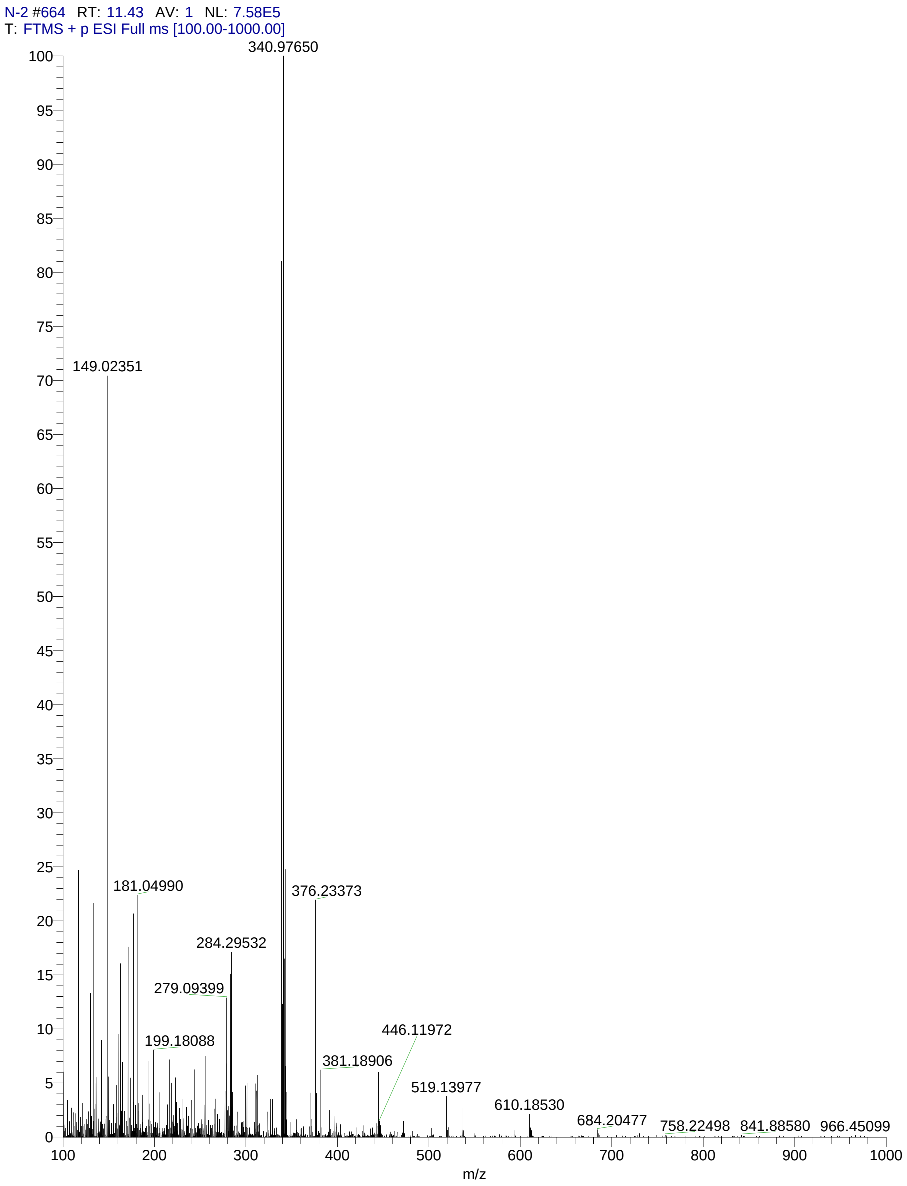

Figure S1. Base peak chromatograms of ingredients from LBLF by UPLC-MS in ESI+ (A), and the MS spetra of peak (B).

Table S1. KEGG term and pathway enrichment analysis of differentially expressed genes between 150 μM H_2O_2 at 24 h treatment and control/LBLF.

NO.	Name (CK-HP)	Map	Count1	Count2	Count3	Count4	p
1	Transcriptional misregulation in cancer	map05202	6	385	52	14225	0.004342267
2	Chronic myeloid leukemia	map05220	3	132	55	14478	0.016278665
3	MAPK signaling pathway	map04010	5	433	53	14177	0.02913758
4	Cytokine-cytokine receptor interaction	map04060	4	296	54	14314	0.030702059
5	Ribosome biogenesis in eukaryotes	map03008	3	186	55	14424	0.038749824
6	Signaling pathways regulating pluripotency of stem cells	map04550	3	186	55	14424	0.038749824
7	Sulfur relay system	map04122	1	9	57	14601	0.038857338
8	Notch signaling pathway	map04330	2	77	56	14533	0.038988457
9	Ether lipid metabolism	map00565	2	79	56	14531	0.040796513
10	Wnt signaling pathway	map04310	3	212	55	14398	0.053233231
11	Prolactin signaling pathway	map04917	2	93	56	14517	0.05429638

NO.	Name (HP-LBLF)	Map	Count1	Count2	Count3	Count4	p
1	ECM-receptor interaction	map04512	2	104	21	14541	0.011855578
2	Neuroactive ligand-receptor interaction	map04080	3	321	20	14324	0.013630615
3	MAPK signaling pathway - fly	map04013	1	195	22	14450	0.026628998
4	Jak-STAT signaling pathway	map04630	2	227	21	14418	0.049521104
5	Nitrogen metabolism	map00910	1	32	22	14613	0.050521625
6	PI3K-Akt signaling pathway	map04151	3	549	20	14096	0.053694086
7	Insulin signaling pathway	map04910	2	242	21	14403	0.055450058
8	HIF-1 signaling pathway	map04066	2	275	21	14370	0.069321833
9	Calcium signaling pathway	map04020	2	280	21	14365	0.071516417
10	Pentose phosphate pathway	map00030	1	53	22	14592	0.081390502
11	Purine metabolism	map00230	2	310	21	14335	0.085152609

Control group (CK), 150 μM H_2O_2 treatment group (HP), and 150 μM H_2O_2 and 100 μg/mL LBLF cotreated group (LBLF), respectively.
